# ABA importers ABCG17 and ABCG18 redundantly regulate seed size in *Arabidopsis*

**DOI:** 10.1111/tpj.70096

**Published:** 2025-03-01

**Authors:** Yuqin Zhang, Moran Anfang, James H. Rowe, Annalisa Rizza, Zhuorong Li, Ning Su, Hamutal Bar, Laurence Charrier, Markus Geisler, Alexander M. Jones, Eilon Shani

**Affiliations:** 1 School of Plant Sciences and Food Security, Tel Aviv University, Tel Aviv, 69978, Israel; 2 College of Advanced Agricultural Sciences, University of the Chinese Academy of Sciences, Beijing 100101, China; 3 Sainsbury Laboratory, University of Cambridge, Cambridge CB2 1LR, UK; 4 Institute of Genetics and Developmental Biology, Chinese Academy of Sciences, Beijing 100101, China; 5 Department of Biology, University of Fribourg, CH-1700 Fribourg, Switzerland

## Abstract

The stress hormone abscisic acid (ABA) plays a crucial role in mediating plant responses to the environment and regulating plant development. In this study, we demonstrate that two ABA importers, ABCG17 and ABCG18, control seed size by regulating the ABA levels transported into the embryo. Double knockdown of *ABCG17* and *ABCG18* resulted in lower ABA accumulation in the embryo, wider siliques, and increased overall seed size. Leaf phloem-specific ABA induction in the *aba2-1* background showed that ABA could move from the vasculature to control seed size. *ABCG17* and *ABCG18* are expressed in leaves, and the reproductive organs septum, and valves but not in the developing seeds, suggesting that ABCG17 and ABCG18 affect seed size maternally. Together, the results shed light on the molecular mechanisms by which ABA is transported to the embryo to determine seed size.

## Introduction

The plant hormone abscisic acid (ABA) is a stress-related signaling molecule^1,2,3,4^. In addition, ABA participates in multiple stages of plant growth and development, including seed development^5,6,7^, dormancy^8^, maturation, and germination^9,10,11^. Current models suggest that there are two ABA accumulation peaks during *Arabidopsis* seed development^8,11,12^. The first ABA maximum occurs during early seed maturation at around 9 days after pollination (DAP).

It is thought that this ABA accumulation is mainly driven by maternal tissues^8,10^. The second ABA accumulation peak occurs at 12 DAP, and high levels of ABA are maintained until the late stages of seed development (21 DAP)^9,13,14^. This maxima affects seed dormancy during the desiccation phase of seed development, and ABA is likely produced by the embryo during this phase^9^. There are two considerable drops in ABA levels during seed maturation. The first takes place around 4 DAP, at the initiation of endosperm cellularization, and is thought to result in larger *Arabidopsis* seeds^11^. The second ABA drop takes place between 9 and 12 DAP, between the end of the embryo growth stage and the beginning of the seed desiccation stage^11^.

Seed size is an essential trait that influences plant growth and development, as well as the yield of crops^15,16^. Several genes and pathways have been identified as regulators of seed size in *Arabidopsis* including the ubiquitin-proteasome system, hormones, and several transcription factors^15,17,18,19^. The ubiquitin-proteasome system is a major regulator of protein degradation in eukaryotic cells. In *Arabidopsis*, mutations in genes encoding components of the ubiquitin-proteasome pathway have been shown to affect seed size. For example, mutations in the RING-type E3 ligase gene, BIG BROTHER, result in abnormally large seeds due to the accumulation of cell wall proteins in the endosperm^20^. G-protein-mediated and mitogen-activated protein kinase-mediated signaling also regulate grain size^21,22,23^.

Like ABA, the plant hormones gibberellins (GAs) play important roles in seed size determination. GAs promote seed growth, whereas ABA can promote or inhibit growth, depending on its concertation^24,25^. The balance between these two hormones is, therefore, critical for seed size regulation^26,27^. While the direct effect of GA on seed size is not entirely clear, mutations in genes involved in ABA synthesis, such as ABA DEFICIENT 2 (ABA2), lead to larger seeds^11,27,28,29^. In addition, the expression of *ABI5* (ABA-INSENSITIVE5) is reduced in *aba2-1* plants, and the *abi5* mutant exhibits larger seeds^11^.

ABA function is regulated at multiple levels including biosynthesis, metabolism, perception and signal transduction, and transport. Since 2010, several ABA transporters from different families have been characterized, shedding light on mechanisms of ABA homeostasis, transport, and redistribution. The first two identified ABA transporters were *Arabidopsis* ATP-binding cassette (ABC) family proteins ABCG25 and ABCG40^30,31^. ABCG25 is a plasma membrane-localized ABA transporter involved in intercellular ABA signaling. It is mainly expressed in the vasculature and controls stomatal movement. Overexpression of ABCG25 leads to higher leaf temperature, suggesting that ABA accumulation in the guard cells causes stomatal closure and reduces transpiration. ABCG40 is also a plasma membrane-localized ABA transporter. It is broadly expressed in roots and guard cells of leaves. ABCG40 functions as an ABA importer and regulates stomatal closure^31^. *Kang et al*. showed that in *Arabidopsis* ABCG25 and ABCG31 function as ABA exporters that move ABA out of the endosperm and that ABCG30 and ABCG40 cooperate to import ABA into the embryo, promoting seed dormancy^32^.

Additional ABA transporter genes have been identified in *Medicago truncatula* (*MtABCG20*)^33^, in wheat (*LR34res*)^34^, in rice (*OsPM1*)^35^, in *Arabidopsis* (genes encoding the nitrate transporter/peptide transporter (NPF) proteins)^36,37,38^, and in tomato (the NPF-encoding *SlAIT1.1*)^39^. However, none of these transporters have been shown to modulate seed size or long-distance ABA transport. ABCG17 and ABCG18 were recently characterized as ABA importers that regulate ABA long-distance transport and ABA homeostasis in guard cells and roots^40^. ABCG17 and ABCG18, localized on the plasma membrane, are mainly expressed in the leaf mesophyll cells, where they promote ABA uptake to tune stomatal closure. Upon abiotic stresses, including high salinity, drought stress, and ABA treatment, both *ABCG17* and *ABCG18* are transcriptionally repressed, which results in more free apoplastic ABA, leading to increased ABA movement to guard cells to balance stomatal closure and to the root to modulate lateral root emergence^40^.

The first evidence that long-distance ABA transport is required for the regulation of seed size in plants came from the recent identification and characterization of OsDG1 in rice^41^. OsDG1 is a multidrug and toxic compound extrusion (MATE) type transporter, which has been shown to promote ABA efflux^41^. The *osdg1* mutant has grain-filling defects caused by noticeably reduced starch content in caryopses. Although ABA is synthesized in leaves of both wild-type and *osdg1* mutant seedlings, only wild-type caryopses accumulate leaf-derived ABA, which activates starch synthesis genes^41^. This process is enhanced at above-normal temperatures to impose an impact on seed development.

In *Arabidopsis*, ABA is required to set seed size in plants^5,8,11,15^, but the complete map of ABA synthesis sites, and the biological importance of ABA movement during seed development has not been established. Therefore, it remains unclear if the entire ABA pool is synthesized directly in the embryo, if ABA moves only a few cells from the seed coat, or if ABA is translocated in a long-distance manner to the seeds, or a combination of all three above.

We previously provided evidence that ABCG17 and ABCG18 are ABA importers that redundantly regulate ABA long-distance transport necessary for control of stomatal closure and lateral root emergence^40^. Here, we show that ABCG17 and ABCG18 also regulate seed size. The double *abcg17/18* knockdown (artificial microRNA targeting *ABCG17* and *ABCG18* transcript at the same time, *mir17,18*) leads to reduced ABA accumulation in the embryo and increased seed size. At the reproductive tissues, *ABCG17* and *ABCG18* are mainly expressed in the valves of the embryo. In addition, we showed that phloem-specific ABA induction influences seed size and that cell type-specific phloem activation of *ABCG17* and *ABCG18* results in larger seeds. These results support the hypothesis that ABCG17 and ABCG18 redundantly govern ABA transport to control seed size.

## Results

### *ABCG17* and *ABCG18* double knockdown leads to larger seeds

**Fig. 1:**
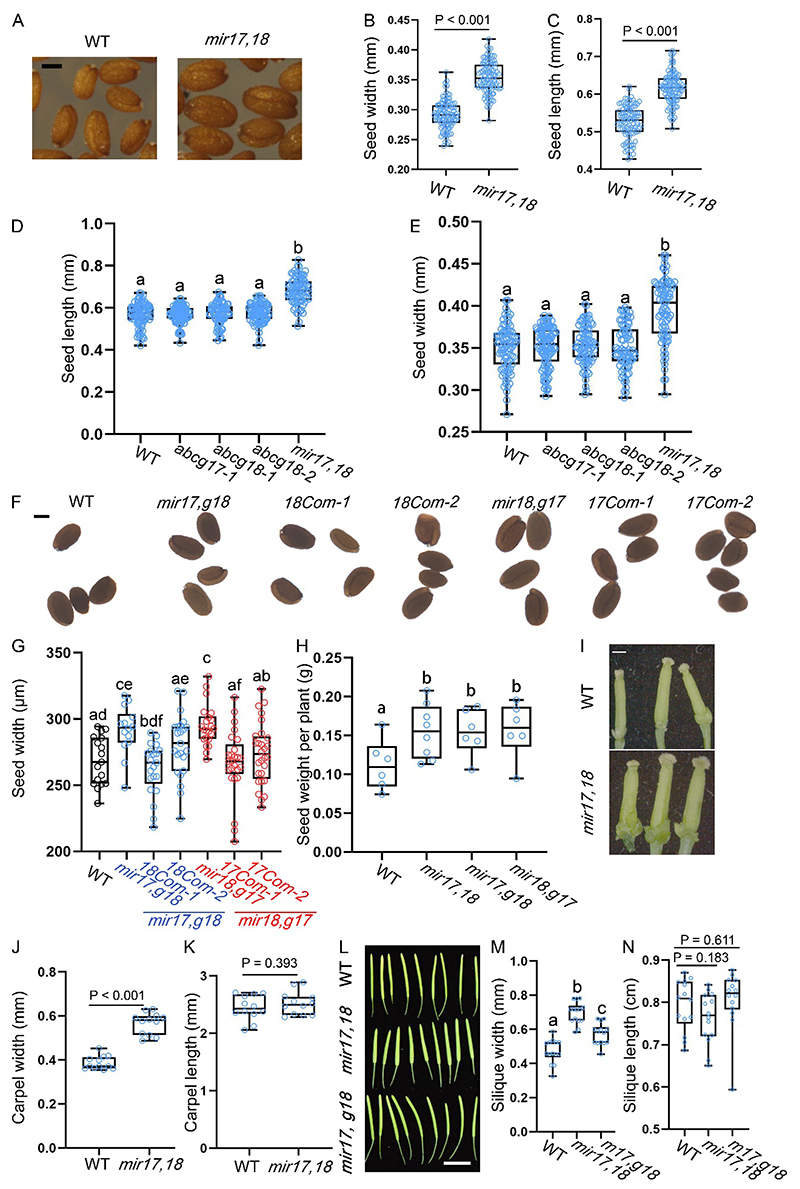
ABCG17 and ABCG18 redundantly regulate seed size. **A**, Light microscopy image of seeds from WT and *mir17,18* plants. Scale bar = 200 μm. **B-C**, Average (±SD) seed B) widths and C) lengths of the indicated genotypes. n ≥ 102; P value determined by Student’s t test. **D-E**, Average (±SD) seed D) lengths and E) widths of the indicated genotypes. n ≥ 80; different letters represent significant differences at P < 0.05, one-way ANOVA with Student’s t test. **F**, Images of seeds from the indicated lines. Com is used to indicate the complementation lines *pABCG17:ABCG17* or *pABCG18:ABCG18*. Scale bar = 200 μm. **G**, Average (±SD) seed widths for WT and indicated complementation lines. n ≥ 14, P < 0.05, one-way ANOVA with Student’s t test. Com stands for two independent complementation lines in the indicated background. **H**, Average (±SD) seed yield per plant for WT and indicated lines. n ≥ 6; different letters represent significant differences at P < 0.05, one-way ANOVA with Student’s t test. **I**, Images of carpels of 35-day-old WT and *mir17,18* plants grown in soil under normal conditions. Scale bar = 0.5 mm. **J-K**, Average (±SD) carpel J) widths and K) lengths of the indicated genotypes. n = 12 plants; P values determined by Student’s t test. **L**, Images of siliques of the indicated genotypes. Scale bar = 1 cm. **M-N**, Average (±SD) silique M) widths and N) lengths of the indicated genotypes. *mir17,18* is *abcg17* and *abcg18* double-knockdown amiRNA line; *mir17,g18* is *mir17* (*amiRNA-ABCG17*) transformed into the background of *abcg18-1* T-DNA insertion line. n ≥ 13; different letters represent significant differences at P < 0.05, one-way ANOVA with Student’s t test.

In a previous study, we demonstrated that ABCG17 and ABCG18 are two PM-localized ABA importers^40^. Since *ABCG17* and *ABCG18* are genetically linked, and obtaining a double mutant through T-DNA crossings is not feasible, the *ABCG17* and *ABCG18* double mutant was generated using an *amiRNA-ABCG17*/*ABCG18* driven by *35S* promoter (*mir17,18*). In addition to a physiologically related guard cell closure phenotype, the double-knockdown *mir17,18* had significantly larger seeds than wild-type (WT) plants. Seeds of *mir17,18* were wider and longer compared to WT (Fig. 1A-C). To test if the larger seed phenotype is caused by the double knockdown of *ABCG17* and *ABCG18*, or by the loss of one of the proteins, we compared the single mutants (*abcg17-1, abcg18-1, abcg18-2)* to *mir17,18* and WT. Although *mir17,18* seeds were significantly larger, the seed size of all single mutants was similar to WT (Fig. 1D-E). *mir17* showed a slight difference in seed width, with no significant difference in seed length (Sup. Fig. 1A). Previous studies showed that ABA is involved in seed size determination^11,15,42^. We, therefore, hypothesized that ABCG17 and ABCG18 control seed size by mediating ABA translocation and accumulation levels during seed development.

To validate that seed-size phenotypes result from the double knockdown of *ABCG17* and *ABCG18*, we tested the seed-size phenotype in two independent knockdown lines. *mir17,g18* is the *mir17* (*amiRNA-ABCG17*) transformed into the background of the *abcg18-1* T-DNA insertion line. *mir18,g17* is the *mir18* (*amiRNA-ABCG18*) transformed into the background of the *abcg17-1* T-DNA insertion line. Both lines were previously described^40^. amiRNA transformation into T-DNA insertion line is needed since *ABCG17* and *ABCG18* are genetically linked. Seeds of both mutant lines were significantly wider than seeds from WT plants (Fig. 1F-G). In addition, we used native promoters that drive *ABCG17* or *ABCG18* to test for rescue of a T-DNA insertion mutant^40^. Except for one *ABCG18* line (*18Com-2*) that showed intermediate rescue, all other lines showed significant rescue of seed-size phenotypes (Fig. 1F-G), confirming that *ABCG17* and *ABCG18* are both necessary for the regulation of seed size. Furthermore, we found that while the seed number per silique was reduced in the *mir17,18* double mutant (Sup. Fig. 1B-C), the seed mass per plant was significantly higher in the double-knockdown combinations compared to the WT (Fig. 1H).

We also evaluated carpel widths in the double-knockdown lines. Interestingly, *mir17,18* lines showed thicker carpel widths but no significant difference in carpel lengths compared to WT carpels (Fig. 1I-K). Since the *Arabidopsis* silique is formed by the fusion of two carpels^2^, we evaluated silique widths and lengths and found that *mir17,18* and *mir17,g18* double-mutant plants had wider siliques but no significant difference in silique length compared to WT (Fig. 1L-N). These data indicate that ABCG17 and ABCG18 redundantly balance seed and carpel development.

### *ABCG17* and *ABCG18* are expressed in valves and funiculus

**Fig. 2:**
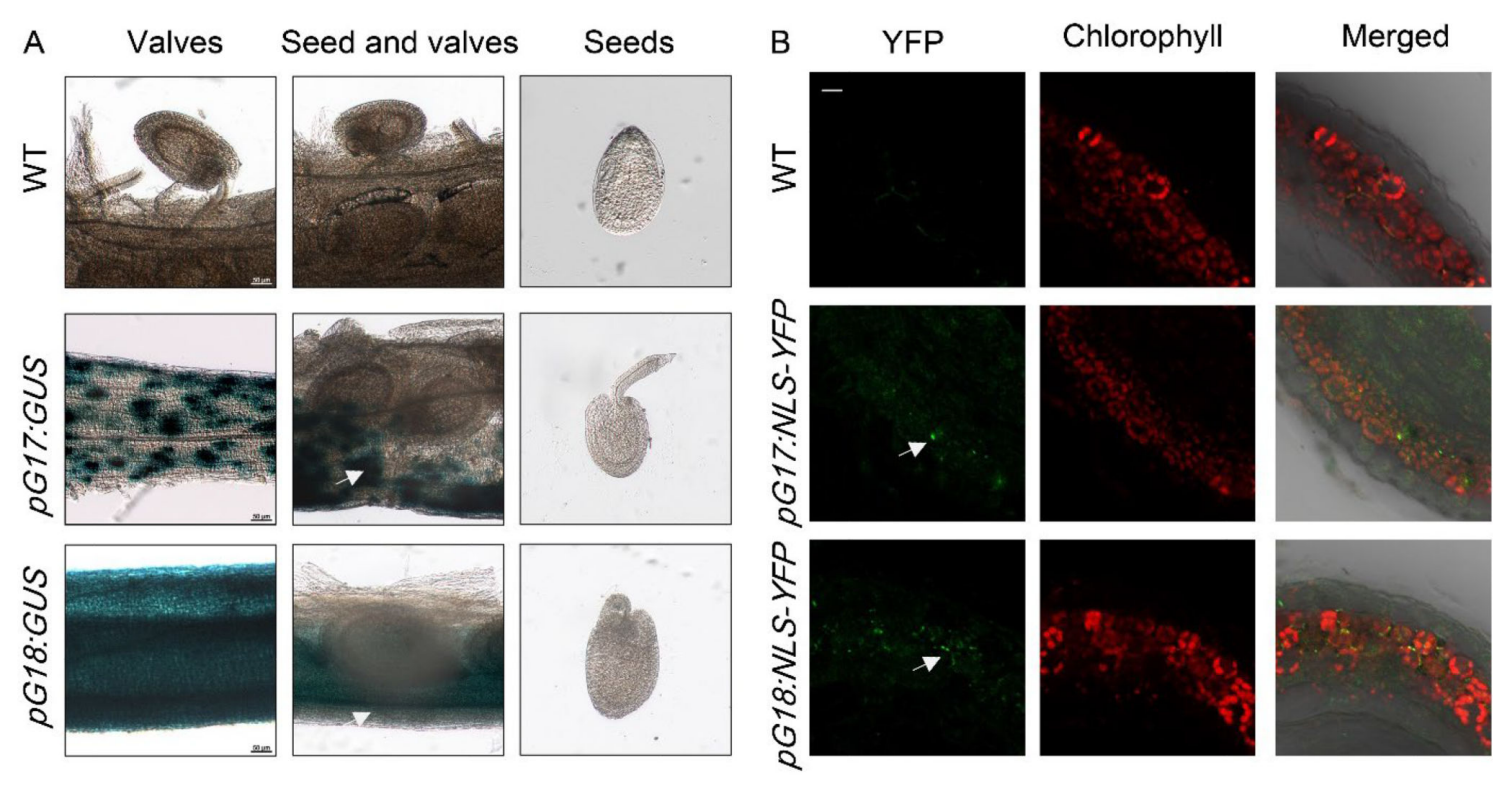
*ABCG17* and *ABCG18* are primarily expressed in valves during the early stage of seed development but not in the embryo or endosperm. **A**, Images of *pABCG17:GUS* (*pG17:GUS*) and *pABCG18:GUS* (*pG18:GUS*) reporters in siliques stained for GUS activity (blue) at 4 DAP. Scale bars = 50 μm. **B**, Images of *pABCG17:NLS-YFP* (*pG17:NLS-YFP*) and *pABCG18:NLS-YFP* (*pG18:NLS-YFP*) reporters in siliques at 4 DAP. YFP signal (green) is detected in valves. Chlorophyll autofluorescence in red. Scale bar = 10 μm. White arrows indicate the signal in the valves.

We next characterized the expression patterns of the two ABCGs in siliques at 4 DAP using *β*-glucuronidase (GUS) reporter lines. In *pABCG17:GUS* and *pABCG18:GUS* lines, imaging of GUS indicated that *ABCG17* and *ABCG18* were primarily expressed in the valves, but not in the ovules (Fig. 2A). Analyses of YFP signal in cross-sections of siliques of *pABCG17:NLS-YFP* and *pABCG18:NLS-YFP* plants at 4 DAP were consistent with results obtained from the GUS lines with valves (Fig. 2B, Sup. Fig. 2A). No expression was detected in the developing seeds (i.e., embryo or endosperm). Notably, it is possible that expression in different tissues is below our detection level. Data from the eFP browser indicate that *ABCG18* is expressed in testa during early seed developmental stages (no expression indicated for *ABCG17)*. The *Arabidopsis* RNA-seq database (ARS) that integrates publicly available *Arabidopsis* RNA-seq library data^43^ showed a high similarity to our GUS and YFP reporters, with undetectable expression in the developing seeds (Sup. Fig. 2B).

**Fig. 3:**
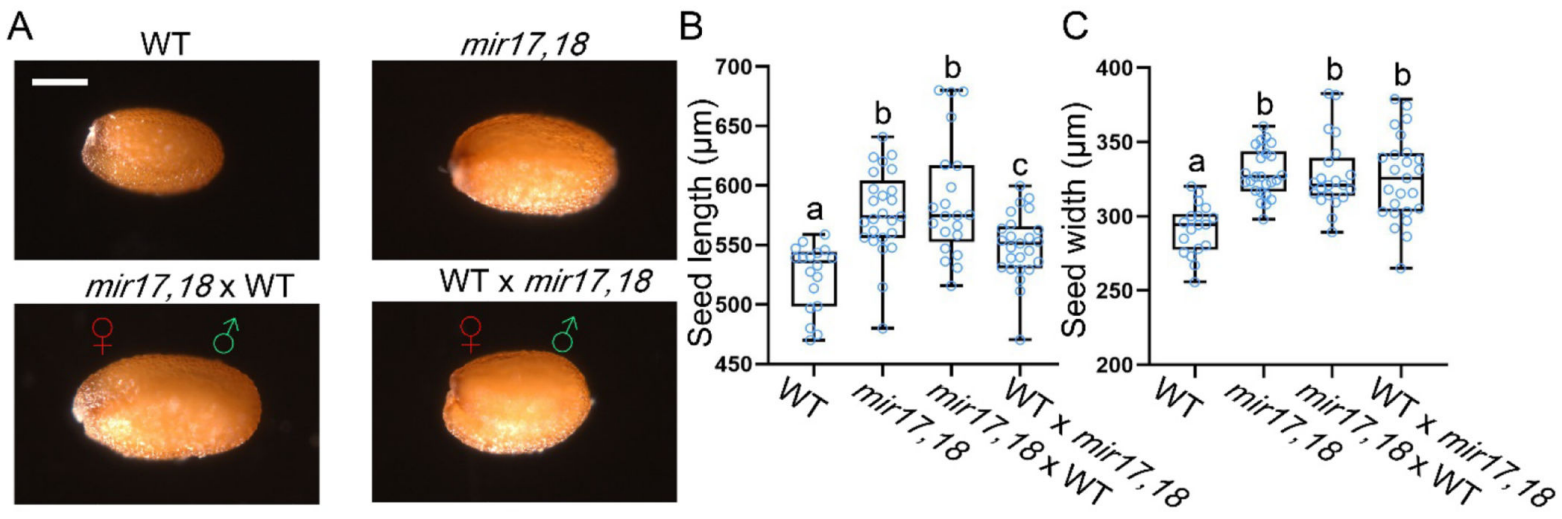
Seed size is affected by both maternal and zygotic ABCG17 and ABCG18 activity. **A**, Images of mature seeds reciprocally crossed with *mir17,18* and WT plants. Scale bar = 200 μm. **B-C**, Average (±SD) seeds B) lengths and C) widths of WT and *mir17,18* reciprocally crossed lines. n ≥ 18; different letters represent significant differences at P < 0.05, one-way ANOVA with Student’s t test. ♀ stands for female and **♂** stands for male.

The results indicate that ABCG17 and ABCG18 adjust seed size, while we cannot detect the expression of these genes in the developing seeds. We, therefore, speculated that ABCG17 and ABCG18 control ABA movement from the maternal region of the plant to the developing seeds. To assess this hypothesis, we tested whether the *ABCG17, ABCG18* double-knockdown seed-size phenotype is influenced by maternal genotype. Seed size was examined in F1 reciprocal paternal/maternal crosses between WT and *mir17,18*. The larger seed size was observed when *mir17,18* functioned paternally, but seed size was intermediate when *mir17,18* functioned maternally (i.e., from the zygote) (Fig. 3A-C). The maternal effect of ABCG17 and ABCG18 on seed size is consistent with the observed expression of *ABCG17* and *ABCG18* in maternal tissues and suggests that ABCG17 and ABCG18 affect seeds from maternal tissues. Notably, a smaller but significant paternal effect was quantified (mean seed size 95% of WT), suggesting a partial contribution of ABCG17 and ABCG18 from the zygote itself.

### ABCG17 and ABCG18 double-knockdown leads to lower ABA levels in the embryo

In order to test how ABA accumulation is affected at the spatial level by *ABCG17* and *ABCG18* knockdown, we utilized the ABA reporter *pRAB18:GFP*^44^. The *pRAB18:GFP* reporter is not expressed in the seeds (i.e., embryo or endosperm). However, we found that *ABCG17* and *ABCG18* double knockdown resulted in enhanced *pRAB18:GFP* signal from the valves compared to WT (Sup. Fig. 3). This supports our hypothesis that ABCG17 and ABCG18 mediate ABA transport into seeds probably from the values.

**Fig. 4:**
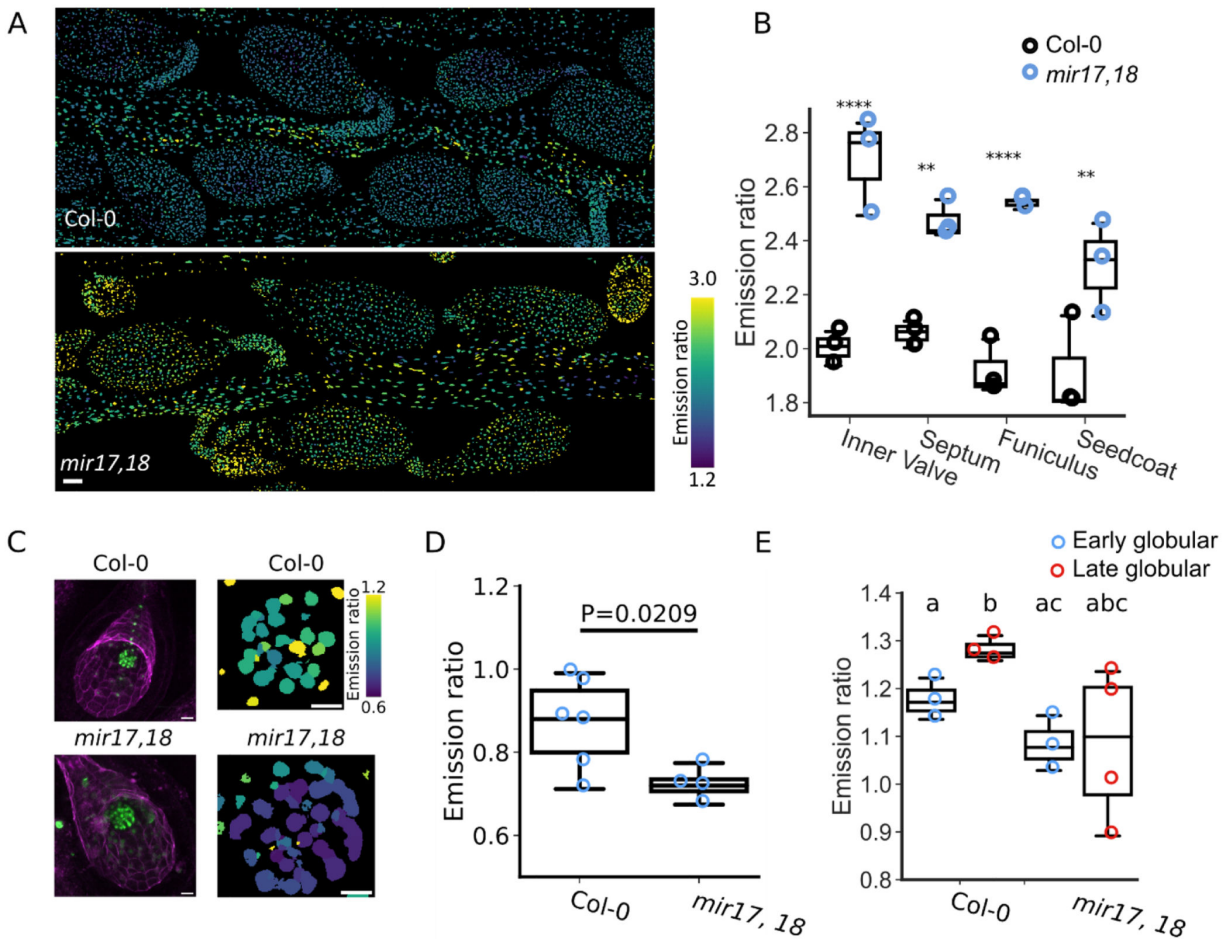
ABA levels are lower in *abcg17,18* double knockdown embryos than in WT, but higher in valves, septum, funiculi and seed coat. **A**, Maximum Z- projection of emission ratios of developing *nlsABACUS2-100n mir17,18* siliques. Siliques were staged for 4 DAP from F1 plants (*ABACUS2* as the male), then hand dissected to reveal the inner valve tissues, septum, funiculi and seeds. Scale bar indicates 50 μm. **B**, Emission ratios of developing inner valve, septum, funiculi and seed coat, as represented in A. The FRETENATOR ROI Labeller was used to label tissues and points indicate the mean emission ratio of each tissue type within a Z-stack. n = 3; two-way ANOVA (Tissue: p = 0.034, genotype: p < 0.0001. Interaction: p = 0.137) with Sidak’s test, **: P < 0.01, ****: P < 0.0001. **C**, Z-sum projection of *nlsABACUS2-400n* fluorescence (green) and autofluorescence (magenta) of F1 *nlsABACUS2-400n* X Col-0 or F1 *nlsABACUS2-400n* X *mir17,18* seeds at 4 DAP (*ABACUS2* as the male). Right: Emission ratios of embryos cropped from images to the left. Scale bars = 20 μm. **D**, Emission ratios of cropped early globular embryos, as represented in C. n ≥ 5; P value determined by Student’s t test. **E**, Emission ratios of cropped early (blue) and late (red) globular embryos. Points indicate the median emission ratio of individual embryo Z-stacks. n ≥ 3; different letters represent significant differences at P < 0.05, one-way ANOVA with Student’s t test.

To better understand ABA levels in the developing seeds, we used the FRET-based ABACUS2-400n and ABACUS2-100n ABA reporters, which enables quantitative, high-resolution analysis of ABA levels in plants^45^. Imaging the silique (4 days after pollination) showed high ABA levels in the outer tissues of the developing seeds, the valves, funiculus, septum and seed coat in *ABCG17* and *ABCG18* double-knockdown compared to WT (Fig. 4A-B). On the contrary, there was a significant reduction in ABA levels in the *ABCG17* and *ABCG18* double-knockdown embryos compared to WT embryos (Fig. 4C-E). The low levels of ABA in the mutant embryo, together with the larger seed size in the *ABCG17* and *ABCG18* double-knockdown lines are in agreement with previous reports showing that the ABA biosynthesis mutant *aba2-1* and the ABA signaling mutant *abi5* develop larger seed^11^. Altogether, the data suggest that ABCG17 and ABCG18 reduce seed size by regulating ABA levels from maternal tissues into the embryo.

### Ectopic production of ABA in the phloem increases seed size

**Fig. 5:**
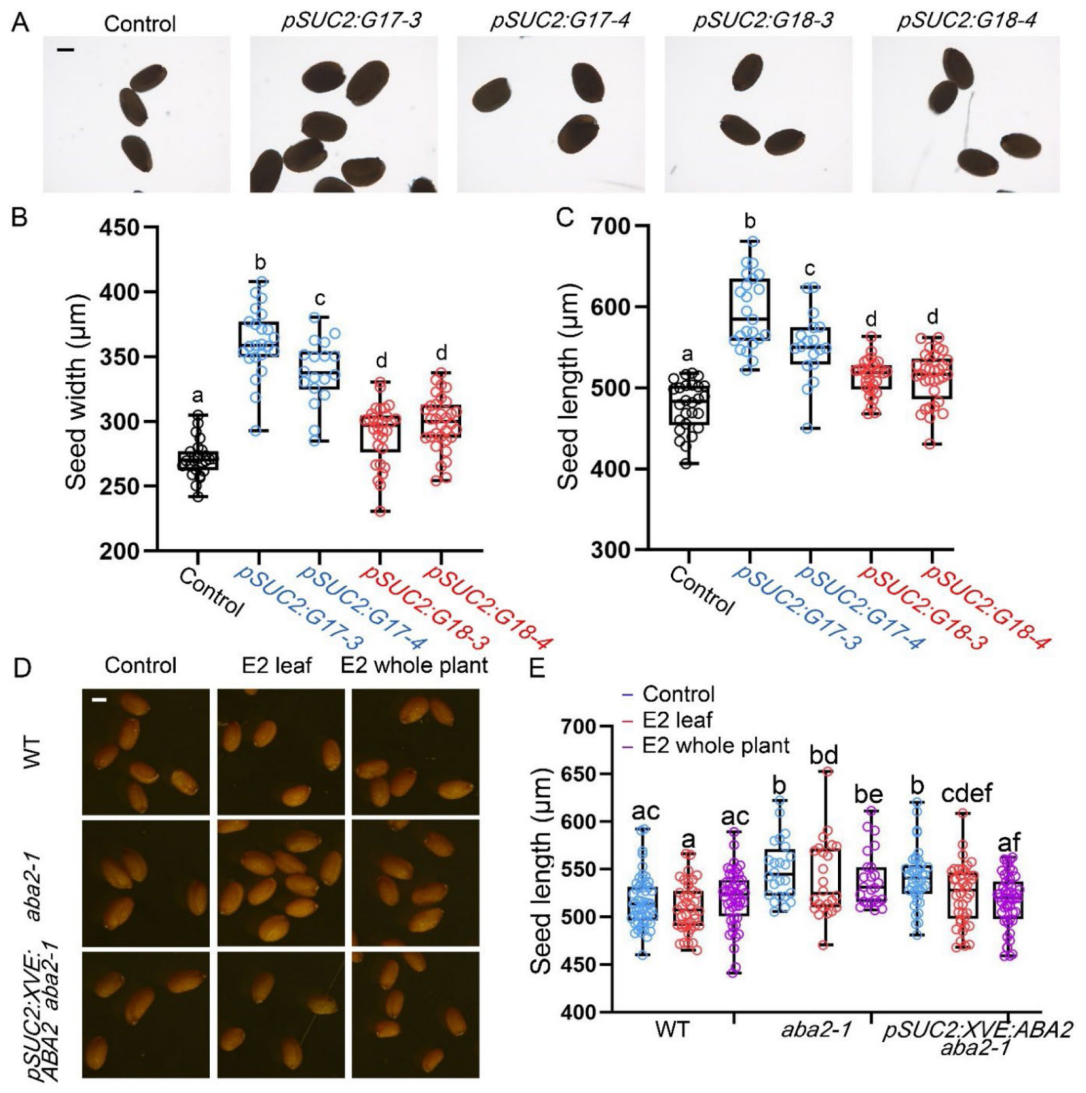
Phloem-born ABA regulates seed size. **A**, Images of seeds from WT plants and from the indicated genotypes that express *ABCG17* or *ABCG18* only in phloem, in the background of *pRAB18:GFP* reporter. Scale bar = 200 μm. **B-C**, Average (±SD) of seeds B) length and C) width of the indicated genotypes. n ≥ 18; different letters represent significant differences at P < 0.05, one-way ANOVA with Student’s t test. **D**, Images of seed length of the WT (Col-0), *aba2-1* mutant and *pSUC2:XVE:ABA2 aba2-1* (*pSUC2:XVE:ABA2* plants inducibly express *ABA2* specifically in phloem companion cells, in the *aba2-1* mutant background). The three genotypes were treated with the following treatments: mock-treated (Control), β-estradiol (E2) applied specifically to rosette leaves; and β-estradiol (E2) applied to the whole plant. Scale bar = 250 μm. β-estradiol (E2) concentration = 5 μM, plants were sprayed 3 times a week until the seeds were harvested. **E**, Quantification of the images shown in (D). Average (±SD) of seed length from β-estradiol (E2) treatment only to the leaves; whole plant, or mock-treated (Control) plants of the indicated genotypes. Estradiol concentration = 5 μM. n ≥ 24; different letters represent significant differences at P < 0.05, one-way ANOVA with Student’s t test.

Our data suggest that ABCG17 and ABCG18 redundantly promote the movement of ABA from maternal tissues into the embryo. To test if ABCG17 and ABCG18 can affect ABA transport from phloem to seeds, we generated *pSUC2:XVE:ABCG17* and *pSUC2:XVE:ABCG18* lines, in which expression of the transporter is driven by a phloem-specific *pSUC2:XVE* promoter. The XVE activator is strictly regulated by estradiol, with limited activity in the absence of an inducer. We could not detect *SUC2* expression in the developing seed (Sup. Fig. 4), suggesting that it is a valuable tool for this experiment. *pSUC2:XVE:ABCG17* and *pSUC2:XVE:ABCG18* plants were shorter than WT plants (Sup. Fig. 5) and produced larger seeds compared to WT and their respective controls (Fig. 5A-C). These results imply that the expression of either *ABCG17* or *ABCG18* manipulates ABA levels and affects leaf size.

To further test the role of phloem-born ABA in affecting seed size, we generated *pSUC2:XVE:ABA2 aba2-1* lines, in which *ABA2* expression is driven by a phloem-specific *pSUC2:XVE* promoter (an estradiol-induced system) in the *aba2-1* mutant background. Importantly, no *SUC2* expression was detected in the developing seed (Sup. Fig. 4). We found that, without the application of estradiol, *pSUC2:XVE:ABA2 aba2-1* displayed seed elongation phenotype, mirroring the phenotype observed in *aba2-1* mutants. However, estradiol treatment only to the rosette leaves, or to the entire plant, suppressed the *aba2-1* phenotype to WT levels (Fig. 5D-E). Therefore, phloem-specific ABA synthesis is sufficient to complement the *aba2-1* mutant seed phenotype, suggesting that ABA can move from the phloem to the developing seeds.

### ABCG1 is an ABA-induced plasma membrane-localized transporter

**Fig. 6:**
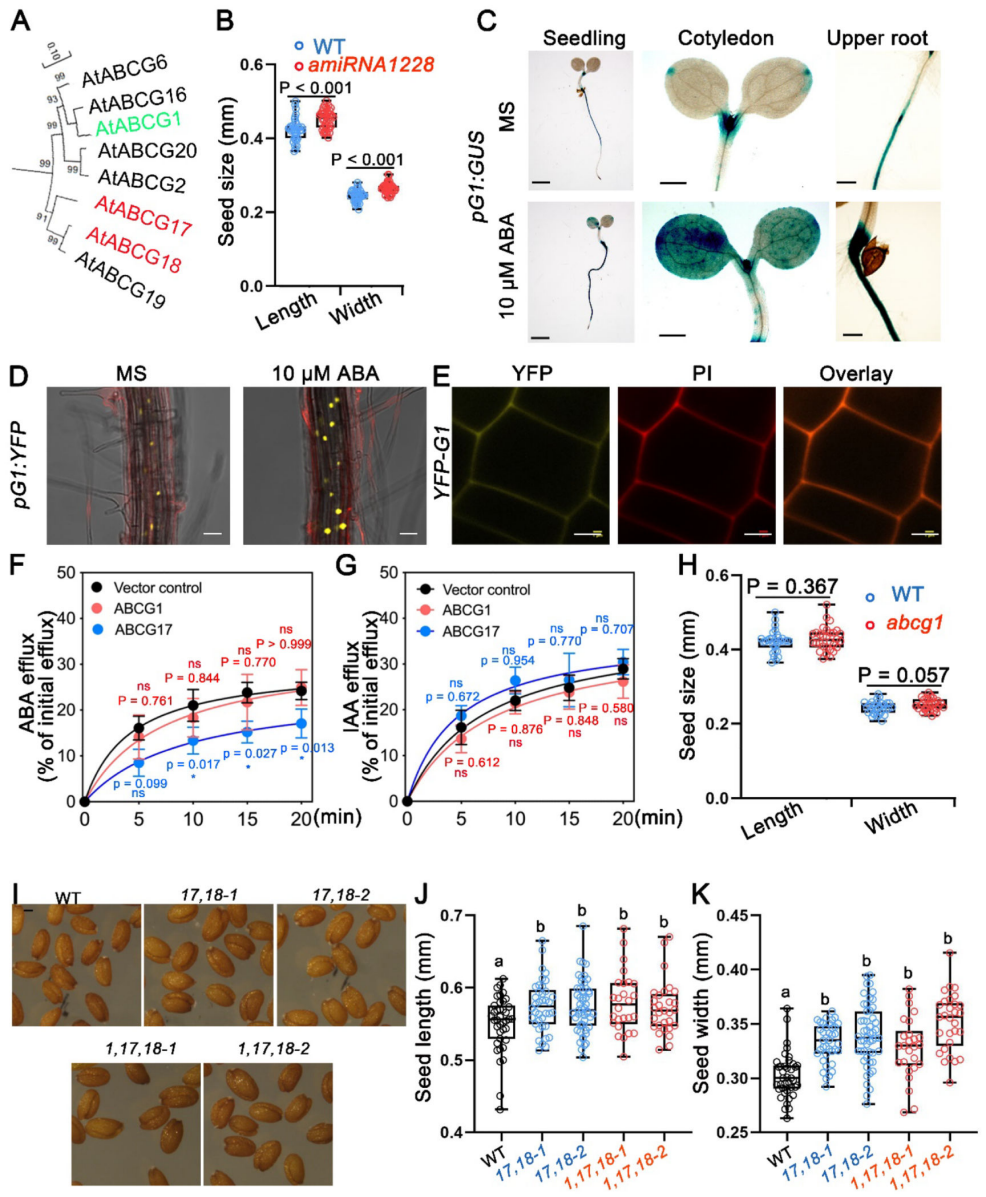
ABCG1 is transcriptionally induced by ABA and localized to the plasma membrane. **A**, Phylogenetic clade of ABCG17, ABCG18, and their close *Arabidopsis* paralogues. **B**, Average (±SD) of length and width of seeds from WT (blue) and a*miRNA1228* line. n ≥ 40; P was determined by one-way ANOVA with Student’s t test. **C**, Images of GUS staining for *pABCG1:GUS* (*pG1:GUS*) 5-day-old seedlings grown with or without 10 μM ABA. Scale bars = 1 mm. **D**, Representative images of *pABCG1:NLS-YFP* (*pG1:NLS-YFP*) in roots treated or not with 10 μM ABA for 7 hours. Scale bars = 10 μm. **E**, ABCG1 subcellular localization in *p35S:YFP-ABCG1* (*YFP-G1*) stable transgenic *Arabidopsis* lines. Yellow indicates YFP-ABCG1 fluorescence, and red indicates propidium iodide (PI). Fluorescence was imaged in the root meristem epidermis layer. Scale bars = 5 μm. **F-G**, Relative efflux of F) [^3^H]ABA and G) [^14^C]IAA from tobacco protoplasts, expressing ABCG1 or ABCG17, relative to initial efflux. n ≥ 4, P values determined by Welch’s *t* test. **H**, Averages (±SD) of seed length and seed width of *abcg1* mutant. n ≥ 30; P values determined by Student’s t-test. **I**, Images of seeds from WT, two triple knockdown lines resulting from *mir18* transformation into *abcg1,17* double mutant. Scale bar = 250 μm. **J-K**, Average (±SD) of length and width of seeds from indicated genotypes. n ≥ 26; different letters represent significant differences at P < 0.05, one-way ANOVA with Student’s t-test.

*ABCG17* or *ABCG18* were originally identified in an amiRNA screen^40^. In the *amiRNA-1228* line, expression of *ABCG17, ABCG18*, and *ABCG1* are reduced (Fig. 6A) as shown previously^40^. The seeds of the *amiRNA-1228* mutant are significantly larger than those of WT plants (Fig. 6B). ABCG1 has not been reported to be involved in ABA transport or activity. To test if *ABCG1* is expressed in the seed, we generated *pABCG1:GUS* lines and discovered that *ABCG1* is weakly expressed in developing seeds (Sup. Fig. 6A). We also generated a *pABCG1:NLS-YFP* line, and its analysis confirmed that *ABCG1* is minimally expressed in developing seeds (Sup. Fig. 6B). We further tested the *ABCG1* transcriptional response to ABA treatment; both *pABCG1:GUS* and *pABCG1:NLS-YFP* lines showed an enhanced response to ABA treatment in both shoot and root (Fig. 6C-D). To determine where ABCG1 localizes at the subcellular level, we cloned the coding sequence of *ABCG1* tagged with the *YFP* coding sequence at the N-terminus driven by the *35S* promoter. Confocal microscopy of the root meristem of the *p35S:YFP-ABCG1* transgenic line indicated that ABCG1 is localized to the plasma membrane (Fig. 6E).

To test if ABCG1 can transport ABA, a biochemical transport assay with radiolabeled ABA ([^3^H]ABA) was performed in tobacco plants that overexpress ABCG1. Unlike the positive control ABCG17, ABCG1 did not show an export activity that was significantly different from the control. Further, ABCG1 did not transport a radioactively labeled hormone from the auxin class ([^14^C]IAA) (Fig. 6F-G). While the results may suggest that ABCG1 is not an ABA transporter, it could be that the protein is not functional under this experimental setup (e.g., codon usage, miss-localization in tobacco protoplasts, or that the YFP fusion alters its activity). Notably, ABCG1 was shown to be involved in the transport of longer-chain aliphatic monomers from the cytoplasm to the apoplastic space during root suberin formation^46^, a process that is induced by ABA^38,46, 47,48^.

To evaluate the contribution of ABCG1 to seed-size phenotype, we examined the *abcg1* mutant; seeds were similar in length and width to WT seeds (Fig. 6H). In addition, when we transformed *mir18* into the double-mutant *abcg1,17* background to generate the triple mutant *m18,g17,g1*, we found that ABCG1 did not alter seed size compared to the double mutant (Fig. 6I-K). While it could be that ABCG1 is not directly involved in seed size development, it is possible that its activity is masked by additional unknown genetic factors or physical constraints. Together, these data indicate that ABCG1 is a plasma membrane protein that is transcriptionally upregulated by ABA treatment.

## Discussion

Our previous study showed that ABCG17 and ABCG18 are two plasma membrane-localized ABA importers that redundantly modulate stomatal closure and lateral root initiation^40^. Here we further characterized the functions of these two importers and found that the double-knockdown lines had larger seeds compared to the WT and single *abcg17* and *abcg18* mutants, suggesting the two ABCG ABA transporters function redundantly to modulate seed development and seed size.

Given that the double knockdown lines showed higher ABA accumulation in the valves, septum, funiculus and seed coat, but reduced ABA content in the embryo (as shown using *nlsABACUS2*), and that ABCG17 and ABCG18 are ABA importers mainly expressed in the valves of 4 DAP siliques, we hypothesized that the two ABCG proteins are redundantly indispensable for ABA accumulation in the zygote while the seeds are in early stage of development. We speculate that ABCG17 and ABCG18 act from the valves, which reduced ABA accumulation in the developing seeds of the *ABCG17* and *ABCG18* double mutant leading to a delay in endosperm cellularization, resulting in larger seeds.

At present, the exact mechanisms governing the accumulation of abscisic acid (ABA) in plant valves remain unclear. Specifically, it is uncertain whether ABA in the valves is synthesized locally or transported from the vasculature of photosynthetic leaves, or if both processes contribute to its presence. ABCG17 and ABCG18, two important ABC transporters, are known to be expressed in the mesophyll cells^40^, where they function to import ABA into these cells. This import process serves to regulate ABA availability, limiting its concentration in the guard cells and roots. In the *ABCG17 ABCG18* double mutant, elevated ABA levels are observed in the guard cells and roots, leading to enhanced stomatal closure and a suppression of later root emergence. Interestingly, while these transporters appear to control ABA levels in guard cells and roots, the same double mutant shows lower ABA levels in the embryo, suggesting that the transporters play a pivotal role in modulating ABA distribution within the plant. This raises the possibility that ABA does not simply passively flow through the phloem to accumulate in the seeds but instead is actively regulated and gated by the activities of ABCG17 and ABCG18. However, the contribution of these transporters in the valves remains an open question—specifically, whether their expression in these tissues has a direct impact on seed size. Is the ABA level in the seed governed by transporter activity within the rosette leaves, or does it involve regulation in the reproductive organs? Moreover, the role of additional transporters in ABA transport within the seeds has yet to be fully elucidated. It is still unclear how the transport of ABA in the seed is coordinated to maintain homeostasis between the zygote and endosperm, two key components of seed development. Further research into the interactions between these transporters and other potential ABA transporters will be critical to understanding the intricate regulation of ABA in seed development and its broader role in plant physiology.

Plant ABCG transporters are renowned for their versatility, particularly through a phenomenon known as “multispecificity.” This term refers to the ability of these transporters to selectively translocate a limited number of substrates, which are often chemically diverse. Unlike the polyspecificity observed in organisms such as yeast, plant ABCGs exhibit a more targeted and refined specificity^49^. For instance, Nicotiana tabacum’s ABCG1/PDR1 plays a role in pathogen defense by selectively translocating cyclic diterpenes like sclareol, manool, and cembrane, while excluding other monoterpenes such as eucalyptol^50^. Similarly, *Arabidopsis* ABCG37/PDR9 demonstrates multispecificity by exporting both the auxin precursor indole-3-butyric acid (IBA) and scopoletin, a compound involved in iron uptake^51, 52,53^. Additionally, Medicago truncatula’s ABCG46 exemplifies multispecificity by transporting structurally distinct precursors of the phytoalexin medicarpin, including p-coumaric acid and liquiritigenin^54^. These examples underscore the critical role of multispecificity in regulating plant growth, defense, and metabolism, ultimately shaping the plant’s dynamic interaction with its environment. In this instance, ABCG1 has been shown to participate in the transport of long-chain aliphatic monomers, a key process in regulating root suberin formation. Here, we demonstrate that ABCG1 is not directly involved in seed size or abscisic acid (ABA) transport, further contributing to the ongoing discussion surrounding the multispecificity of the ABCG family. Similar specificity was shown for the ABA importer ABCG40 and the ABA exporter ABCG25, with both exhibiting specificity towards ABA enantiomers^30,31,55^.

It will be important to investigate if the activity of rice DG1, which facilitates ABA long-distance transport to control rice seed development in a temperature-dependent manner^41^, is maintained in *Arabidopsis* and other dicots. This might be a challenging task since the MATE transporter family is large, with complex redundancy between family members^56,57^. If DG1 activity is indeed conserved, studying the relationships between the two ABA transport mechanisms will be interesting. These two activities could complement each other to stream ABA from the leaves to the developing seeds. Alternatively, they might function in response to different environmental stimuli with DG1 mediating high-temperature responsive ABA transport^41^ and ABCG17 and ABCG18 meditating ABA transport in response to abiotic stress^40^. Interestingly, it was recently reported that the rice ABA biosynthesis mutant *nced3* (*Osnced3*), produces smaller grains while the *OsNCED3* overexpression lines generated larger grains^58^, opposite to *Arabidopsis* ABA2, suggesting for low mechanistic conservation or complex regulation.

Recently, three NPF transporters, NPF2.12, NPF2.13, and NPF2.14, were found to regulate GA and ABA translocation in *Arabidopsis* to promote endodermal root suberization^38^. Though these three transporters are very close in the phylogenetic tree, NPF2.12 and NPF2.13 are localized on the plasma membrane and function as ABA importers, whereas NPF2.14 is localized on the tonoplast and functions as an ABA exporter in oocytes. Further, NPF2.12 and NPF2.13 are required for long-distance GA precursor GA_12_ shoot-to-root translocation^38^. This raises the question of whether the ABA intermediates and conjugated forms (e.g., Abscisic acid glucosyl ester) are those that are transported long and short distances^59^.

Previous studies have shown that diverse plant hormones, including, auxin, ABA, cytokinin, brassinosteroid, and GA, play critical roles in regulating seed size ^21,5,60,61,62,63,64^. It was also reported that ABA biosynthesis and signal transduction regulate seed development^10^, as well as DG1-mediated activity of ABA transport to balance rice grain filling^41^. However, the spatial activity map within the seeds that orchestrate the zygote and endosperm ABA homeostasis and communication is not completely clear. By carrying out *in situ* hybridization and GUS staining experiments, *Cheng et al*. found that a key enzyme in ABA biosynthesis pathway, *ABA2*, is expressed in both embryo and endosperm, suggesting that ABA may be synthesized directly in these tissues^11,19^. However, we demonstrated here that maternal ABA is transported to the embryo and determines seed size.

How does ABA regulate seed size? An earlier study showed that there is a correlation between the timing of endosperm cellularization and final seed size, with delayed cellularization resulting in larger seeds^11^. Further, ABA negatively modulates endosperm proliferation^5,11, 65,66,67,68^. Our study showed reduced ABA accumulation in the embryo in the *ABCG17* and *ABCG18* double-knockdown plants. It will be interesting to investigate if there is a delay in endosperm cellularization in this mutant. At this point, the relationship between the ABA levels in the embryo and seed size is not clear. Does ABA directly synchronize cell division and expansion in the endosperm, or rather it affects seed size indirectly by orchestrating nutrient sugar allocation is not fully understood. It will be important to understand in the future the complete ABA spatiotemporal map during seed development and reveal the direct mechanisms downstream to ABA perception that control seed size.

## Materials and methods

### Plant materials and growth conditions

All *Arabidopsis thaliana* lines used in this work are in Col-0 background (Columbia ecotype from Salk Institute). For assays on plates, sterilized seeds were plated on 16 × 16 cm square Petri dishes or 8.5-cm round Petri dishes with growth media containing 0.5× Murashige-Skoog (MS) medium, 1% sucrose, and 0.8% plant agar, pH 5.7. The seeds were stratified for 2-3 days at 4 °C and then transferred to growth chambers (Percival, CU41L5) at 21 °C and 100 μE m^−2^ S^−1^ light intensity under long-day conditions (16 h light/8 h dark). For seed propagation, transformation, crossing, or soil pot assays, seeds were sown into wet soil, and the plants were grown in growth rooms under long-day conditions (16 h light/8 h dark) at 21 °C. Sequencing data for *Arabidopsis* genes used in this study can be found in the *Arabidopsis* Genome Initiative database under the following accession numbers: *ABCG1* (*AT2G39350*), *ABCG17* (*AT3G55100*), and *ABCG18* (*AT3G55110*). *pSUC2:YFP* was obtained from Gregory Vert Lab^69^.

### Agrobacterium transformation

The GV3101 electrocompetent *Agrobacterium tumefaciens* strain was incubated on ice with ~100 ng of plasmid for 2 min and electroporated using a MicroPulser (Bio-Rad Laboratories; 2.2 kV, 5.9 ms). Immediately after electroporation, 600 μl LB medium was added, and the samples were shaken for 2 h at 28 °C. The Agrobacteria were then plated on selective LB agar plates containing the relevant antibiotics for 2 days at 28 °C.

### *Arabidopsis* transformation

An *Agrobacterium* colony was chosen and verified by colony PCR and sequencing before growth in 150 ml LB medium containing 25 μg/ml gentamycin and 50 μg/ml rifampicin plus construct specific antibiotic for 2 days at 28 °C. Agrobacteria were collected by centrifuging for 15 min at 1790 g (4000 rpm). The supernatant was discarded, and the pellet was resuspended in 60 ml 5% sucrose and 0.05% Silwet L-77. *Arabidopsis* flowers were then submerged in the bacterial solution for around 5 min. After this, plants were kept in the dark overnight and then grown until siliques ripen and dried. T1 seeds were collected in bulk and sown on MS media containing the appropriate antibiotics for transformant plant selection. Resistant plants were transferred to soil and grown until maturity for seed collection.

### Genotyping

T-DNA insertion lines for single mutants ordered from Gabi Kat (www.gabi-kat.de) and The Arabidopsis Information Resource (www.arabidopsis.org/) are listed in Table S1. Primers for the T-DNA insertion mutant genotyping were designed using the T-DNA Primer Design Tool powered by Genome Express Browser Server (http://signal.salk.edu/tdnaprimers.2.html). Homozygous mutants were characterized by PCR carried out with primers listed in Table S2.

### Cloning

*mir17,18; mir17,g18* and *mir18,g17* lines, were previously described^40^. *ABCG1, ABCG17*, and *ABCG18* coding regions were amplified using Phusion High-fidelity Polymerase (New England Biolabs) from Col-0 complementary DNA (cDNA) using primers listed in Table S3 (cloning of *ABCG17* and *ABCG18* were previously reported^40^). Promoters of *ABCG1, ABCG17*, and *ABCG18* were amplified from Col-0 DNA using Phusion High-fidelity Polymerase (New England Biolabs) using primers listed in Table S3 (cloning of *ABCG17* and *ABCG18* promoters was previously reported^40^). Promoters of *ABCG1, ABCG17*, and *ABCG18* are 1759, 1276, and 1580 base pairs long, respectively, including the 5′ untranslated regions. *ABCG1, ABCG17*, and *ABCG18* coding regions as well as their promoter fragments were cloned into pENTR/D-TOPO (Invitrogen K2400), verified by sequencing, and subsequently cloned into binary destination vectors using LR Gateway reaction (Invitrogen 11791). *p35S:YFP-ABCG1, p35S:YFP-ABCG17*, and *p35S:YFP-ABCG18* were generated using the pH7WGY2 vector and were selected using spectinomycin in *Escherichia coli* and hygromycin in plants. *p35S:ABCG1, p35S:ABCG17*, and *p35S:ABCG18* were generated using the pH2GW7 vector and selected using spectinomycin in *E. coli* and hygromycin in plants. *pABCG1:NLS-YFP, pABCG17:NLS-YFP*, and *pABCG18:NLS-YFP* were generated using R1-R2:NLS-YFP in the pART27 vector and selected using spectinomycin in *E. coli* and kanamycin in plants. *pABCG1:GUS, pABCG17:GUS*, and *pABCG18:GUS* were generated using the pWGB3 vector and were selected using kanamycin and hygromycin in *E. coli* and hygromycin in plants. Using primers containing the recognition sequence for the restriction enzyme AscI, *pABCG17* and *pABCG18* were amplified and ligated into *ABCG17* CDS and *ABCG18* CDS at the AscI site. The LR Gateway reaction was carried out with the binary vector pGWB1. *pABCG17:ABCG17* and *pABCG18:ABCG18* were transformed into *mir18,g17* and *mir17,g18-1*, respectively, for phenotype complementation assays. To generate *pSUC2:XVE:ABA2:NosT*, a multisite Gateway reaction (LRII+) was carried out: *pSUC2:XVE* (in p1p4r position) with *ABA2* CDS (in pENTR) and NosT terminator (in 2R3e position) was cloned into pB7m34GW. The WMD3 website was used to design amiRNAs (http://wmd3.weigelworld.org/cgi-bin/webapp.cgi) (Table S4). The amiRNA sequences were synthesized by Syntezza Bioscience Ltd. and were cloned into the pUC57 vector with Gateway system borders and then into the pH2GW7 destination vector using the Gateway system.

### Plant genetics

All transgenic lines generated from the destination vectors were transformed into Col-0 with the exception of *pSUC2:XVE:ABA2CDS:NosT*, which was transformed into the *aba2-1* mutant. T1 seeds were collected and selected on 1/2 MS plates containing appropriate antibiotics or on soil with basta spraying. At least 10 independent lines for each construct were generated. Characterization of two representative homozygous lines for each construct are reported.

### Seed-size measurements

Images of the seeds were taken using a light microscope. The seed length and seed width were quantified using Fiji software.

### Estradiol treatments

β-estradiol (E2758, Sigma Aldrich) was dissolved in ethanol at 10 mM and stored at −20 °C. 5 μm β-estradiol as the final working concentration with 0.01-0.02% Tween 80 was used in the β-estradiol treatment assays. 10 days-old seedlings were treated with estradiol (5 μm) after transplanting to the soil. Estradiol was applied 2 times a week until siliques matured (turned yellow). The experiment included three groups: 1. Control (mock-treated plants); 2. Estradiol treatment only on the leaves, (no flowers and siliques); and 3. Estradiol treatment to the entire plant.

### GUS staining and histology

Histochemical detection of GUS activity was carried out using 5-bromo-4-chloro-3-indolyl-D-glucuronide as a substrate as previously described^70^. Samples were placed on slides with glass coverslips and imaged with a Zeiss binocular microscope. Histology of GUS-stained samples was performed using protocol described previously^71^.

### Radioactive ABA translocation and transport assays

[^3^H]ABA (ART2192; 1 mCi/ml and 10 Ci/mmol) and [^14^C]IAA (ARC0160; 0.1 mCi/ml and 55 mCi/mmol) export from tobacco (*Nicotiana benthamiana*) mesophyll protoplasts were analyzed as described^72^. Tobacco mesophyll protoplasts were prepared 4 days after agrobacterium-mediated transfection with *p35S:YFP-ABCG1* or empty vector as a control. Relative export from protoplasts was calculated from exported radioactivity into the supernatant as follows: Percent export = (radioactivity in the supernatant at time t = x min) − (radioactivity in the supernatant at time t = 0) × (100)/(radioactivity in the supernatant at t = 0 min). Values presented are means from eight ([^3^H]ABA) and four ([^14^C]IAA) independent transfections.

### FRET confocal imaging

UBQ10pro::nlsABACUS2-400n (line7) pollen was used to pollinate emasculated Col-0 or *mir17,18* flowers. At 4 days after crossing, siliques were embedded in 5% agarose (w/v), and sectioned with a Leica VT1200S vibratome (Leica Biosystems, section width: 100 μm). Samples were mounted in sterile deionized water and imaged immediately. An upright SP8-Fliman was used for nlsABACUS2-400n imaging. All images were acquired as Z-stacks in 16 bit mode, with a 20× dry 0.70 HC PLAN APO dry objective. Typically, sequential scanning was used with the following laser/detector settings: Sequence 1 was 442 excitation 50%, HYD1 460-500 nm, 200 gain and HYD2 525-550 nm, 200 gain. Sequence 2 was 514 excitation 30%, HYD2 525-550 nm, 200 gain and PMT2 650-750 nm, gain 650. Other parameters were offset 0; scan speed 400; line accumulation 2-4; bidirectional X on; pinhole 1 airy unit; Z-step size equal to the optical section thickness; zoom 2.45; resolution 800 x 800 pixels.

### Biosensor image analysis

Embryos were cropped with Fiji and then analyzed with FRETENATOR: Segment and ratio (v2.0 alpha, https://github.com/JimageJ/FRETENATOR2) to quantify emission ratio as described previously^73,74^. Local label-based background subtraction was used to remove autofluorescence from images.

### Statistical analysis

Multiple comparisons were performed by using one-way ANOVA with the least significant difference post hoc test in SPSS 19.0. Two-tailed Student’s t tests were used for graphs with only two groups. Statistical significance was determined at P < 0.05 unless otherwise stated.

## Supplementary Material

Supplementary Data
