# Supplementary material for: ABA importers ABCG17 and ABCG18 redundantly regulate seed size in Arabidopsis
Source: Plant J. Author manuscript; Available in PMC 2025 Apr 4. (PMC7617562; doi:10.1111/tpj.70096)
Supplement: Supplementary Data [file EMS203671-supplement-Supplementary_Data.pdf]

ABA importers ABCG17 and ABCG18 redundantly regulate seed size in *Arabidopsis*

Yuqin Zhang[1,2,✉], Moran Anfang[1], James H. Rowe[3], Annalisa Rizza[3], Zhuorong Li[2], Ning Su[2,4], Hamutal Bar[1], Laurence Charrier[5], Markus Geisler[5], Alexander M. Jones[3], Eilon Shani[1,✉]

[1] School of Plant Sciences and Food Security, Tel Aviv University, Tel Aviv, 69978, Israel.

[2] College of Advanced Agricultural Sciences, University of Chinese Academy of Sciences, Beijing 100101, China.

[3] Sainsbury Laboratory, University of Cambridge, Cambridge B2 1LR, UK.

[4] Institute of Genetics and Developmental Biology, Chinese Academy of Sciences, Beijing 100101, China.

[5] Department of Biology, University of Fribourg, CH-1700 Fribourg, Switzerland.

✉ Corresponding author

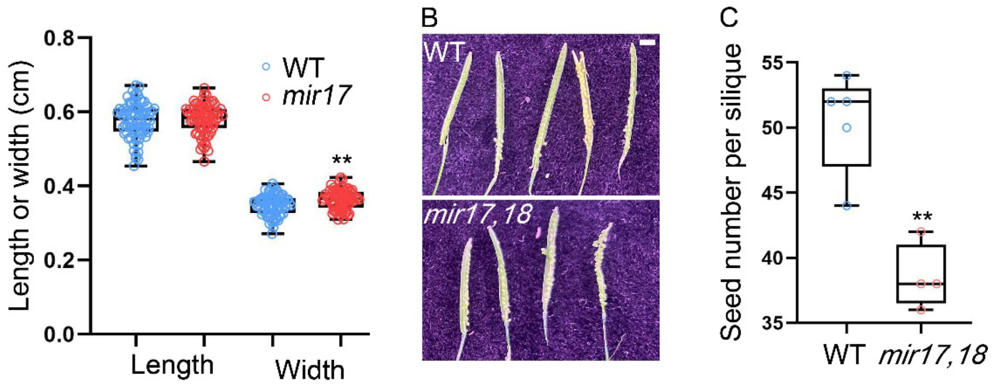

Sup. Fig. 1. Double knockdown of *ABCG17* and *ABCG18* contains reduced seed number per each silique compared to WT. A, Seed length and width of the indicated genotypes. n ⩾ 73. P value < 0.01, Student's t test. **B,** Images of siliques of the indicated genotypes. Scale bar = 1 mm. **C,** Average (±SD) seed number of each silique of the indicated genotypes. n ≥ 4, **, P value < 0.01, Student's t test.

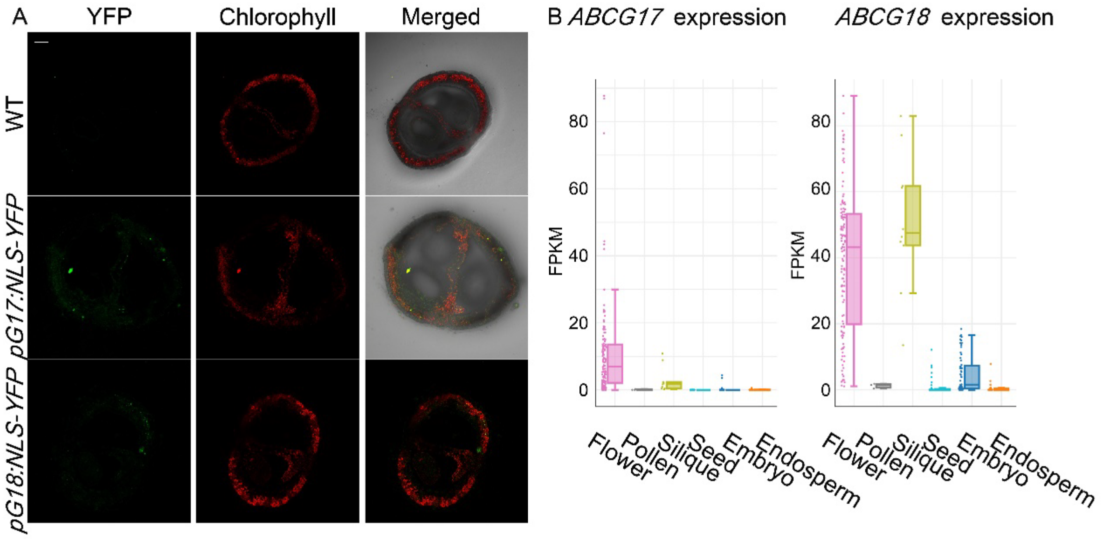

Sup. Fig. 2. *ABCG17* and *ABCG18* are expressed in valves in the early seed developing stage. **A**, Images of *pABCG17:NLS-YFP* (*pG17:NLS-YFP*) and *pABCG18:NLS-YFP* (*pG18:NLS-YFP*) reporters in siliques at 4 DAP. YFP signal (green) is detected in valves. Chlorophyll autofluorescence in red. Scale bar = 50 µm. **B**, Expression levels of *ABCG17* and *ABCG18* at different tissues, obtained from online RNA-Seq data (http://ipf.sustech.edu.cn/pub/athrna/). FPKM stands for Fragments Per Kilobase of transcript per Million mapped reads.

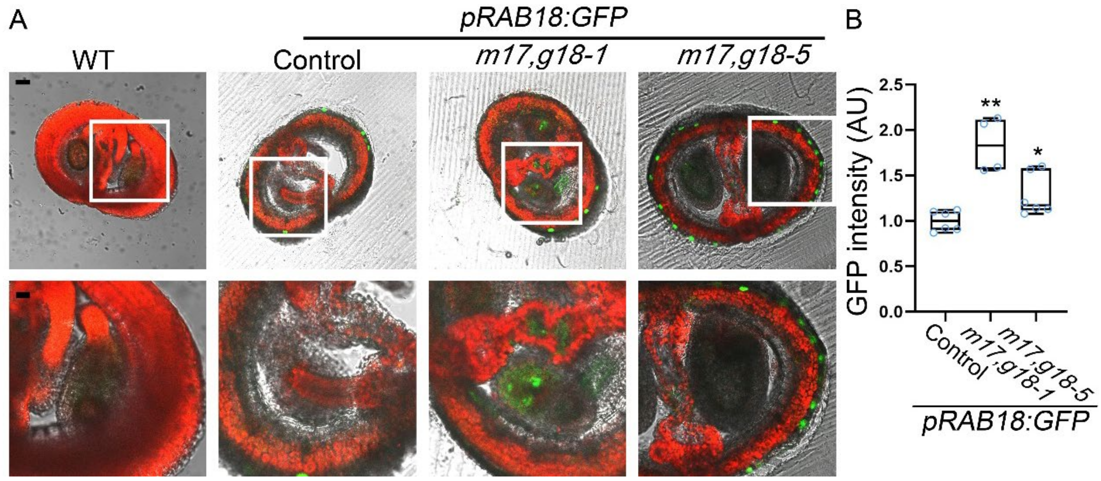

Sup. Fig. 3. Double knockdown of *ABCG17* and *ABCG18* leads to changes in ABA response in the valves. A, *pRAB18:GFP* signal in *mir17,g18* and Control siliques at 4 DAP. *mir17,g18* is *mir17* (*amiRNA-ABCG17*) transformed into the background of *abcg18-1* T-DNA insertion line. Shown are two independent transformation lines (#1 and #5). Green is GFP fluorescent signal, and red is chlorophyll. Scale bar = 50 µm. **B,** Average (±SD) GFP intensities in indicated lines. n ≥ 4; *, P value < 0.05, **, P value < 0.01, Student's t test.

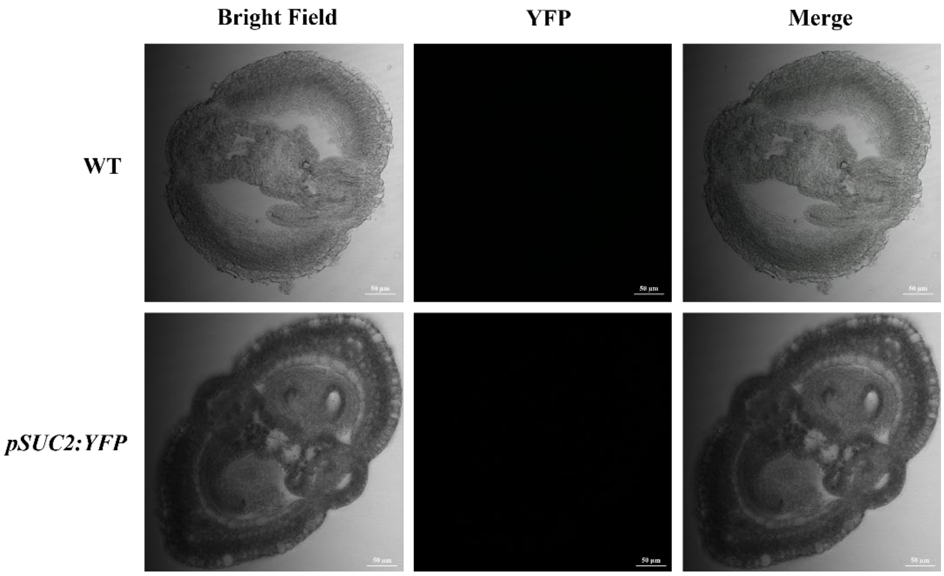

Sup. Fig. 4. ***SUC2*** **is not expressed in the developing seed.** YFP signal, driven by *pSUC2*, is not detected in developing seeds, 4-days after pollination. Scale bar = 50 μm.

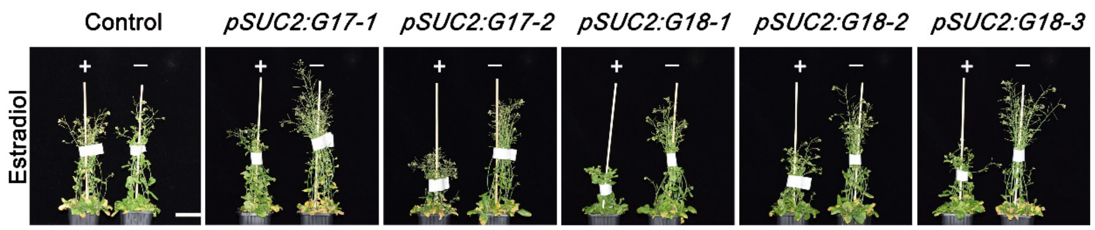

Sup. Fig. 5. Phloem-specific expression of *ABCG17* or *ABCG18* leads to reduced growth. Images of 45-day-old estradiol-treated vs. mock-treated WT, *pSUC2:ABCG17*, and *pSUC2:ABCG18* plants. *G17* and *G18* indicates for *ABCG17* and *ABCG18*. Scale bar = 5 cm. + indicates for 5 µm estradiol treatment, - indicates for mock treatment.

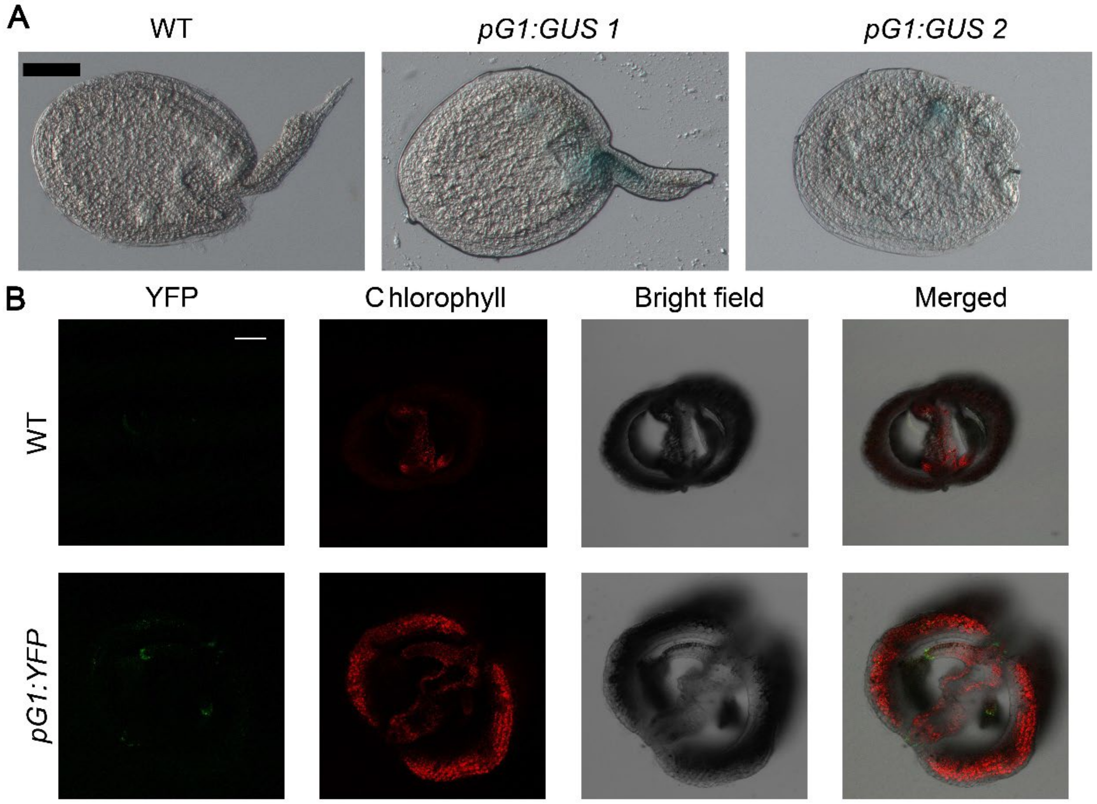

Sup. Fig. 6. *ABCG1* is weakly expressed in the zygote. A, Images of GUS stained WT and *pABCG1:GUS* seeds at 4 DAP. Scale bars = 100 µm. **B,** Images of 4 DAP WT and *pABCG1:NLS-YFP* silique with YFP (*pG1: YFP*) signal in green and chlorophyll in red. Scale bar = 50 µm.

Table S1. T-DNA insertions.

Gene	Gene accession	T-DNA line	Insertion
ABCG17	*AT3G55100*	CS332619	Chr3 20421138
ABCG18	*AT3G55110*	SALK_100187	Chr3 20425486
ABCG1	*AT2G39350*	SALK_061511	Chr2 16431923

Table S2. Primers used for genotyping T-DNA lines.

Gene	Primer name	Primer sequence (5'-3')
abcg17 *CS332619*	*abcg17*-LP	GCAGAACAGCTTCGTAGGGATACT
	abcg17-RP	TGATGCATTAGCAGGACA
	BP	ATTTTGCCGATTTCGGAAC
abcg18-1 SALK_100187	*abcg18-1*-LP	AGAAGAGACCCCAAGCTAACG
	abcg18-1-RP	TCACAGAGTTCGCACTTGATG
	BP	ATTTTGCCGATTTCGGAAC
abcg1 SALK_061511	*abcg1*-LP	GTCAATAAAACCCATTTCGCC
	abcg1-RP	ACTTCTCGGGAGACGAAACTC
	BP	ATAATAACGCTGCGGACATCTACATTTT

Table S3. Cloning primers.

Promoter/gene	Forward primer (5'-3')	Reverse primer (5'-3')
pABCG17	CACCTCACGCCCTCTTATTCTT GCTTCC	TCACGCCCTCTTATTCTTGC TTCC
pABCG18	CACCTCACGCCCTCTTATTCTT GCTTCC	TCACGCCCTCTTATTCTTGC TTCC
ABCG17 CDS	CACCATGCTGCAAAGAGACGC CGT GATC	TCACGCCCTCTTATTCTTGC TTCC
ABCG18 CDS	CACCATGCCACGTGTTTCGGC GGAAATT	TCACGTCCTCTTATTCTTAC TCCC
ABCG1 CDS	CTCCACCGTCATCACCGTA	CAACATCACACTAGGAACC ACAC
pABCG1	GGAGGTGAAGTCCTGCTTTAA TGAGATATGCGAG	CGAGCCATGGCGCGCCCAC CCTTACAATAG

Table S4. *amiRNA* sequences.

Targeted gene	amiRNA sequences
ABCG17	TTATTTGTCCTGCTAACGCAT
ABCG18	TAAGATAAACGTTTCCGGCAA
ABCG17,18	TGTTTAGAGTTACCGTGGCTT
